# HIV diversity and drug resistance from plasma and non-plasma analytes in a large treatment programme in western Kenya

**DOI:** 10.7448/IAS.17.1.19262

**Published:** 2014-11-18

**Authors:** Rami Kantor, Allison DeLong, Maya Balamane, Leeann Schreier, Robert M Lloyd, Wilfred Injera, Lydia Kamle, Fidelis Mambo, Sarah Muyonga, David Katzenstein, Joseph Hogan, Nathan Buziba, Lameck Diero

**Affiliations:** 1 Division of Infectious Diseases, Alpert Medical School, Brown University, Providence, RI, USA; 2 Center for Statistical Sciences, Brown University, Providence, RI, USA; 3 Division of Infectious Diseases, Stanford University, Stanford, CA, USA; 4 Research Think Tank, Inc., Buford, GA, USA; 5 Department of Medicine, School of Medicine, College of Health Sciences, Moi University, Eldoret, Kenya; 6 Academic Model Providing Access To Health Care (AMPATH), Eldoret, Kenya; 7 Department of Hematology and Blood Transfusion, College of Health Sciences, Moi University, Eldoret, Kenya

**Keywords:** HIV, drug resistance, subtype, diversity, Kenya, analyte, AMPATH

## Abstract

**Introduction:**

Antiretroviral resistance leads to treatment failure and resistance transmission. Resistance data in western Kenya are limited. Collection of non-plasma analytes may provide additional resistance information.

**Methods:**

We assessed HIV diversity using the REGA tool, transmitted resistance by the WHO mutation list and acquired resistance upon first-line failure by the IAS–USA mutation list, at the Academic Model Providing Access to Healthcare (AMPATH), a major treatment programme in western Kenya. Plasma and four non-plasma analytes, dried blood-spots (DBS), dried plasma-spots (DPS), ViveST^TM^-plasma (STP) and ViveST-blood (STB), were compared to identify diversity and evaluate sequence concordance.

**Results:**

Among 122 patients, 62 were treatment-naïve and 60 treatment-experienced; 61% were female, median age 35 years, median CD4 182 cells/µL, median viral-load 4.6 log_10_ copies/mL. One hundred and ninety-six sequences were available for 107/122 (88%) patients, 58/62 (94%) treatment-naïve and 49/60 (82%) treated; 100/122 (82%) plasma, 37/78 (47%) attempted DBS, 16/45 (36%) attempted DPS, 14/44 (32%) attempted STP from fresh plasma and 23/34 (68%) from frozen plasma, and 5/42 (12%) attempted STB. Plasma and DBS genotyping success increased at higher VL and shorter shipment-to-genotyping time. Main subtypes were A (62%), D (15%) and C (6%). Transmitted resistance was found in 1.8% of plasma sequences, and 7% combining analytes. Plasma resistance mutations were identified in 91% of treated patients, 76% NRTI, 91% NNRTI; 76% dual-class; 60% with intermediate-high predicted resistance to future treatment options; with novel mutation co-occurrence patterns. Nearly 88% of plasma mutations were identified in DBS, 89% in DPS and 94% in STP. Of 23 discordant mutations, 92% in plasma and 60% in non-plasma analytes were mixtures. Mean whole-sequence discordance from frozen plasma reference was 1.1% for plasma-DBS, 1.2% plasma-DPS, 2.0% plasma-STP and 2.3% plasma-STB. Of 23 plasma-STP discordances, one mutation was identified in plasma and 22 in STP (*p*<0.05). Discordance was inversely significantly related to VL for DBS.

**Conclusions:**

In a large treatment programme in western Kenya, we report high HIV-1 subtype diversity; low plasma transmitted resistance, increasing when multiple analytes were combined; and high-acquired resistance with unique mutation patterns. Resistance surveillance may be augmented by using non-plasma analytes for lower-cost genotyping in resource-limited settings.

## Introduction

Antiretroviral therapy (ART) resistance is a cause and a consequence of treatment failure [1, 2]. Optimizing treatment in resource-limited settings (RLS) is problematic due to limited availability and high cost of new ART regimens and treatment monitoring [3–9]. Surveillance studies to evaluate HIV-transmitted drug resistance (TDR) before ART [10] and to characterize acquired resistance upon treatment failure [11, 12] rely on technology not easily accessible for patient management. Less costly, simpler analytes, including dried blood spots (DBS), dried plasma spots (DPS) and ViveSTs (ST; formerly Sample Tankers^®^), could facilitate ART management [13–18].

Adult HIV prevalence in Kenya (5.6–6.1% in 2012) is the 12th highest worldwide, representing a high health-burden on the country [19–21]. HIV-1 infection is highly diverse with co-circulation of subtypes A (50–80%), D (10–20%) and C (5–15%), and multiple recombinants (10–20%) [22–34]. ART access has significantly increased in Kenya since 2001, with positive patient outcomes [35, 36]. First-line ART has included zidovudine/stavudine, lamivudine and nevirapine/efavirenz, with a recent, still on-going, substitution of tenofovir for stavudine, in keeping with recent World Health Organization (WHO) guidelines [37]. Data on drug resistance (DR) in the country are limited. TDR from mother to child was reported in 29–67% [38, 39], and in adults among 1.1–7.5% in the coast and Nairobi [22, 40–44]. Resistance upon ART failure has only been reported in three studies, one describing 14% resistance in 58 injecting drug users in Mombasa with no available treatment histories [45]; the second in 15% of 100 patients from two clinics in Mombasa and Nairobi as part of a multi-site African study [46]; and the third from our work reporting 94% resistance in 28 patients from Burnt Forest, a rural Academic Model Providing Access to Healthcare (AMPATH) clinic [34].

To gain preliminary insight on the magnitude of resistance in diverse circulating subtypes and its potential impact, we examined HIV diversity, TDR in newly diagnosed patients and acquired resistance in treatment-experienced patients failing first-line ART at AMPATH, a large HIV treatment programme in western Kenya [47]. Genotyping from plasma and non-plasma analytes as alternate options for resistance testing were compared, to investigate alternative monitoring strategies in western Kenya and other RLS.

## Methods

### Study setting

As of March 2013, AMPATH [47–50] provided comprehensive clinical services to 138,736 HIV-positive patients. Of those, 78,064 are in care, and 58,841 receive ART. The Moi Teaching and Referral Hospital (MTRH) clinic, AMPATH's largest, enrolled 26,791 adults, 42% on ART. Patients are managed with an electronic medical record [51] according to protocols based on WHO and AMPATH guidelines. At the time of this study, first-line ART included zidovudine/stavudine, lamivudine and efavirenz/nevirapine.

### Patient enrolment

Between May 2006 and November 2007, two groups of HIV-positive adults attending MTRH were offered enrolment. Inclusion criteria for treatment-naïve patients included: 1) no prior ART and 2) most recent CD4 count>350 or<200 cells/mL, in an attempt to identify recent versus chronic infections. Inclusion criteria for treatment-experienced patients included: 1) on WHO-recommended first-line ART >6 months; 2) ART adherence >50% based on patient-report; and 3) suspected of failing therapy based on a CD4 count drop >25% over six months prior to enrolment, or no increase in CD4 after 12 months of ART.

Consenting patients were enrolled sequentially until the desired number of samples was obtained – about 60 treatment-naïve and 60 experienced, 20% higher than the desired enrolment of 100, to account for sample deterioration. Upon enrolment, patients were interviewed, charts were reviewed for demographic, clinical and laboratory characteristics, and blood was obtained. The study was approved by Lifespan and Moi University ethics review boards.

### Laboratory methods

CD4 (FACSCalibur system; Becton Dickenson, San Jose, CA, USA) and viral load (VL) (Amplicor 1.5; Roche Molecular, Pleasanton, CA, USA) testing were done at the AMPATH reference laboratory, which participates in the United Kingdom Quality Assessment Service and National Institutes of Health Department of AIDS Viral Quality Assurance Programs. Virologic failure and detectable VL were defined as VL>400 copies/mL.

Each sample was prepared as: 1) plasma; 2) DBS; 3) DPS; 4) STB; and 5) STP. Plasmas were shipped on dry-ice to the US, and frozen at −80°C. DBS and DPS were prepared with 100 µL/spot on Whatman 903 Protein-Saver Cards. First-generation STs, a novel dried transportation matrix device [13, 16], were prepared with 1 mL plasma (STP) or blood (STB) per ST. Filter and ST analytes were hood-dried overnight upon initial sample preparation. To increase the number of STP sequences, a second STP batch was prepared in the US from thawed frozen-plasma and dried on driDOC, a prototype drying device, according to manufacturer's instructions. Analyses included sequences from both batches. DBS and DPS were stored with desiccant at room temperature, hand-carried to the US and frozen at −80°C. STs were shipped at room temperature to Research Think Tank (Alpharetta, GA, USA) [52] and stored at ambient room temperature for 10–90 days before evaluating the built-in colour indicator capsule and testing.

For DBS and DPS, spots were placed directly into 9 mL of Nuclisens lysis buffer and agitated for 2 hours at room temperature and RNA extraction completed according to the Nuclisens minMag protocol (Biomerieux, Durham, NC, USA). Genotyping was performed as previously described [53]. Briefly, RNA was reverse transcribed via Life Technologies (Carlsbad, CA, USA) SuperScript III One-Step RT-PCR kit and a second-round PCR was performed using Life Technologies Platinum Taq. PCR products were Sanger sequenced. Sequence assembly was with Sequencher (Gene Codes Corporation, Ann Arbor, MI, USA). Amplification was attempted for all plasma, STP and STB and a portion of DBS (64%) and DPS (37%). For success rate calculations, only the portion of STP (37%) and STB (43%) that had “blue” colour indicators were counted. Such indicators are built into the STs pointing to adequate (blue) or inadequate (pink) drying [54].

ST genotyping was performed as previously described [54], and accomplished by recovering ST-dried samples with version 1 kit supplied eluent buffer. RNA extraction was with QIAmp Viral RNA mini Kit (QIAGEN, USA). Sequences were obtained with TRUGENE^®^ HIV-1 Genotyping System (Siemens Healthcare Diagnostics, Tarrytown, NY, USA).

### Data analysis

Patient-level data included age, gender, previous ART and previous CD4. Sample covariates included time spans between sample acquisition and shipment, and between arrival in the US and genotyping.

Sequence quality control was with SQUAT [55]. Hypermutation and susceptibility analyses were with Stanford Database tools, accessed 1 August 2012 [56]. TDR among treatment-naïve patients was interpreted with the WHO mutation list [57] and compared to the IAS–USA list [58]. Acquired resistance among treatment-experienced patients was with the IAS–USA list. Mixtures were considered mutant. Subtype was derived with REGA version 2.0 [59], with manual bootscan assessment.

For plasma and DBS, multivariable logistic regression analyses were used to examine the relationships between genotyping success, as the dependent variable, and log_10_ VL, the duration between sample acquisition and shipping, the duration between shipping and genotyping, and patient stratum (naïve/experienced). For DPS, STP and STB, the logistic regression analyses included only log_10_ VL and patient stratum as independent variables. The linearity assumption was evaluated in all models as an exploratory step using generalized additive models.

For resistance detection concordance between analytes, resistance-associated amino acids were counted as distinct mutations. Mutations between analyte pairs were compared using McNemar's test.

The number of nucleic acid (NA) differences was calculated for each non-plasma/plasma sequence pair. Both complete and partial NA differences were counted and summarized by analyte type. For each non-plasma analyte, discordance rates between treated and naïve and among subtypes were compared using Poisson regression, fit using generalized estimating equations, an unstructured correlation structure between analyte types and an offset corresponding to the log-length of the sequence overlap in the pair. Outcome was number of discordances and dependent variables were analyte type, treatment status and subtype. Robust standard errors were used to calculate 95% confidence intervals and *p*-values.

## Results

### Patients and genotypes

A total of 122 patients, 62 ART-naïve and 60 ART-experienced, were enrolled. The treatment-naïve group was sequentially enrolled from May to August 2006, after screening 436 patients. Of those screened for the treatment naïve group and not enrolled, 102 were not new to clinic, 114 were treated, 40 did not fit CD4 criteria, 111 missed their appointment, three were missed by a research assistant and four refused.

The ART-experienced group was enrolled from January to November 2007 using clinical and immunological WHO criteria. As previously reported as a result of this study, misclassification of virologic failure using these criteria was high [7]. Of 209 patients who met CD4 enrolment criteria and had VL testing, 60 (29%) had detectable VL, and were included. Specific breakdown of patients screened for the treatment experienced group is not available.

Table 1 shows demographic, clinical and genotype characteristics according to patient stratum. Median age of participants was 35 years, 61% were female, median enrolment CD4 was 182 cells/µL (14%) and median VL 4.6 log_10_ copies/mL. Treated patients were on ART a median 2.4 years, most (88%) with stavudine+lamivudine+nevirapine/efavirenz.

**Table 1:** Demographic, clinical and genotypic characteristics of study cohort^a^

Variable	Naïve: CD4<200	Naïve: CD4>350	Treated and failing	Total
Number	28	34	60	122
Age	34 (22, 55)	34 (19, 68)	37 (20, 64)	35 (19, 68)
Male	12/28, 43%	11/34, 32%	24/60, 40%	47/122, 39%
CD4 count	99 (8, 195)	507 (367, 1984)	153 (8, 719)	182 (8, 1984)
CD4%	8 (1, 30)	25.5 (10, 60)	11 (1, 30)	14 (1, 60)
Viral load (log_10_ copies/mL)	5.1 (3.2, 6.3)	4.3 (2.6, 5.3)	4.4 (3.8, 6.0)	4.6 (2.6, 6.3)
Antiretroviral regimen				
3TC, D4T, NVP			40/59, 68%	
3TC, AZT, NVP			5/59, 8%	
3TC, D4T, EFV			12/59, 20%	
3TC, AZT, EFV			2/59, 3%	
Years on antiretroviral therapy			2.4 (0.92, 5.3)	
Subtype				
A	16/28, 57%	21/30, 70%	29/49, 59%	66/107, 62%
D	5/28, 18%	3/30, 10%	8/49, 16%	16/107, 15%
AD	2/28, 7%	4/30, 13%	6/49, 12%	12/107, 11%
C	4/28, 14%	1/30, 3%	1/49, 2%	6/107, 6%
AC	0/28, 0%	1/30, 3%	2/49, 4%	3/107, 3%
Other^b^	1/28, 4%	0/30, 0%	3/49, 6%	4/107, 4%

a Values are presented as *median (range)* for continuous variables and *n/N*, % for categorical variables;

b AG (1/107, 1%); CD (1/107, 1%); undetermined (2/107, 2%). 3TC, lamivudine; D4T, stavudine; AZT, zidovudine; NVP, nevirapine; EFV, efavirenz.

### Genotyping success

A total of 196 sequences were available for 107/122 (88%) patients, 58/62 (94%) naïve and 49/60 (82%) treated. Genotyping success rates were 100/122 (82%) for plasma; 37 of the 78 attempted DBS (47%); 16 of the 45 attempted DPS (36%); 14 of the 44 attempted field-plasma STP with blue indicators (32%) and 23 of the 34 frozen-plasma STP (68%) (one additional STP sequence was subsequently obtained); and 5 of the 42 attempted STB (12%). Differences in STP success rates may be attributable to sample (fresh/frozen), sample handling or drying methods. No participant had sequences from all five analytes; seven had sequences from four analytes; 16 from three; 36 from two; 48 from one (43/48 plasma); and 15 from none (12%). All intra-patient sequences clustered phylogenetically with high bootstrap values. No hyper-mutation was identified.

For plasma, odds of successful genotyping were higher at higher VL but in a non-linear manner suggesting a threshold at approximately 3.5 log_10_ VL. Odds of successful genotyping with VL>3.5 log copies/mL were 9.5 times the odds for VL≤3.5 log copies/mL (95% CI=1.3–69.6, *p*=0.03). Longer shipment-to-genotyping time (mean 6.0 months, range 4.2–8.9) was also predictive of decreased success, with an odds ratio (OR) of 0.66 per one month longer duration (95% CI 0.46–0.96, *p*=0.03), but sampling-to-shipping time was not predictive of genotyping success. For DBS, higher log_10_ VL (OR=3.4, CI=1.3–9.4, *p*=0.01) was associated with higher success but, unlike plasma, the relationship between log odds of success and log_10_ VL was linear and therefore, a threshold was not examined. Longer sampling-to-shipping time (median 2.9 months, range 0.5–16.1) was also associated with lower DBS success, with an OR of 0.5 per one month longer duration (CI=0.3–0.8, *p*=0.003). Higher VL was also associated with STP genotyping success (OR=3.5 per 1 log_10_ higher VL; CI=1.4–8.8; *p*=0.01), but not with DPS. The pink colour indicator within the ST, which identifies inadequate drying, was highly predictive of genotyping success. The association between genotyping success and VL was not examined for STB because of the small sample size (five sequences).

### HIV diversity

Common subtypes were A (62%; 60% A1 and 2% A2), D (15%) and C (6%), followed by various unique recombinants, including AD/DA/DAD/ADA (11%), AC/CA (3%), CD in (1%), AG (1%) and 2% undetermined (Table 1).

### DR in treatment-naïve patients

**Figure 1:**
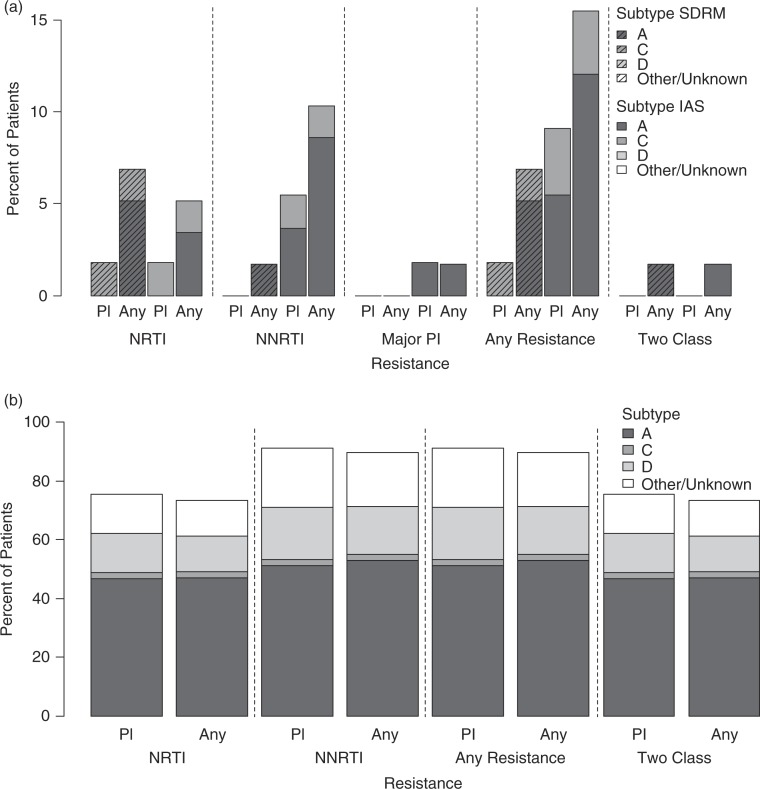
HIV drug resistance in ART naïve (a) and treated (b) patients according to HIV-1 subtype. Bars show percent of patients with transmitted (a) or acquired (b) nucleoside reverse transcriptase inhibitor (NRTI), non-nucleoside reverse transcriptase inhibitor (NNRTI), major protease inhibitor (PI; in (a) only), any and two-class drug resistance. Results for each resistance category are shown for plasma and for any analyte (combining plasma and non-plasma analytes), according to HIV-1 subtype. Results for treatment naïve patients (a) are shown for the World Health Organization surveillance drug resistance mutation (SDRM) list (hashed bars) and for the International Antiviral Society–USA (IAS–USA) list (solid bars). “Pl,” plasma.

Plasma sequences were available for 55/62 (89%), and from any analyte from 58/62 (94%) naïve patients (Figure 1a). Based on the WHO list 1/55 (1.8%, 95% binomial CI 0.04–9.7%) had resistance in plasma (RT L210W; subtype C). However, combining analytes, TDR was observed in 4/58 (7%, CI 1.9–16.7%), including RT T215ST, Y181C (STP; subtype A); D67DN, K219KQ (DPS/STP; subtype A); F77FL (STP; subtype A) and L210LW (plasma/DBS/STP; subtype C). There were no differences in TDR between the two CD4 groups, or among different subtypes.

Using the IAS–USA list, four additional ART-naïve participants, eight in total (14%), had RT resistance; 0/4 in plasma only, 2/4 in non-plasma analytes only and 2/4 combined. One patient had a major PI resistance mutation (Q58E; subtype A) and all had minor mutations, most commonly K20R, M36I, H69K and L89M for subtype A; M36I, H69K and I93L for subtype C; and M36I, I64V and L63P for subtype D. Two-class TDR was seen in 1/58 (1.7%) patients according to either mutation list and 2/58 (3.4%) combining lists.

### DR in treatment experienced patients

Plasma sequences were available for 45/60 (75%) drug-experienced patients and from any analyte for 49/60 (82%) patients (Figure 1b). In plasma, 91% (41/45, CI 78.8–97.5%) had RT-associated mutations, 76% NRTI and 91% NNRTI. No major PI mutations were observed. Fifteen percent had one-class (all NNRTI, CI 6–29%) and 76% dual-class resistance (CI 60–87%). In plasma, the median number of mutations per patient was 4 (range 0–10), 2 (range 0–7) NRTIs and 2 (range 0–5) NNRTIs; 60% had ≥4, and 47% had ≥5 mutations (Table 2). Common NRTI mutations were M184V (76%); T215F/Y (42%); D67N (27%); and M41L (22%). K65R was not observed. Common NNRTI mutations were K103N/S (40%); G190A/S (31%); Y181C (22%); and K101E/H (18%). The number of NRTI, NNRTI or total mutations did not differ by subtype.

**Table 2:** Drug resistance mutation patterns in treated patients failing first-line ART, according to subtype and number of resistance mutations

ID	Subtype	P/NP	NRTI	NNRTI
1	A	1/1	41LM^a^, 67N, 70KR, 75IMV, 184V, 215F, 219KQ	101H, 106M, 190A
2	A	1/0	67N, 70R, 184V, 215F, 219Q	101E, 190A
3	A	1/0	41L, 62V, 184V, 215Y	108I, 181C, 221Y
4	A	1/3	67N, 70R, 184V, 215F, 219E	103NS^b^, 181C
5	A	1/2	67N, 70R, 184V, 219Q^c^	101E, 190A
6	A	1/0	70EK, 116Y, M184V	101E, 181CY, 190A
7	A	1/0	184V, 215FIST	108IV, 181C, 190A, 221Y
8	A	0/2	41L^d^, 184V^d^, 210W^d^, 215Y^d^	103N^d^, 138G^d^
9	A	1/1	75IM, 184V, 215F	103N, 190AG^a^
10	A	1/0	184V, 215F	103N, 138Q, 179L
11	A	1/1	41L^a^, 184V, 215F	103N, 138Q
12	A	1/0	184V, 215F	103N, 106I, 230L
13	A	1/0	41L, 184V, 215Y	103N, 138Q
14	A	1/1	75I, 184V	90I, 181C, 221Y
15	A	1/0	41L, 70R, 184V	188L
16	A	1/0	67N, 184V	101E, 190A
17	A	1/0	67N, 70R, 184V	181C
18	A	1/0	184V, 215Y	101E, 190A
19	A	1/1	184V	103N
20	A	1/0	A62V, M184V	103N
21	A	1/0	184V	181C, 221Y
22	A	0/2	184V^d^	103N^d^
23	A	1/2	184V	190S
24	A	0/1	None^d^	103N^d^
25	A	1/3	None	188L
26	A	1/0	None	103N
27	A	0/1	None^d^	None^d^
28	A	1/0	None	None
29	A	1/0	None	None
30	AC	1/0	41L, 67N, 184V, 210W, 215F	181CY, 190A
31	AC	1/1	41L, 184V, 215Y	98AG^a^, 106IV^a^, 188L
32	AD	1/1	41L^a^, 184V, 215F	103N, 106I, 108I^a^, 221Y^a^, 230L
33	AD	1/2	67N, 70R, 184V, 215F, 219Q	101E, 190A
34	AD	1/0	184V, 215Y	90I, 103N, 138A
35	AD	1/0	67N, 184V	101E, 190A
36	AD	1/0	None	138A
37	AD	1/1	None	None
38	AG	1/2	None	103N
39	C	1/1	41LM^a^, 67N, 70R, 184V, 215F, 219EQ^b^	106M, 179D, 230LM
40	CD	1/1	None	None
41	D	1/1	67N, 184V, 210W, 215Y	108I, 181C, 221Y^c^
42	D	1/0	41L, 67N, 184V, 210W, 215Y	103N
43	D	1/1	184V	103N, 108I, 225H
44	D	1/1	184V	103N, 138GQR
45	D	1/1	184V	103S, 190A
46	D	1/0	184V	190A
47	D	1/1	None	103KN^a^
48	D	1/0	None	103N
49	?	1/0	None	181CY

The table is sorted by overall number of mutations in descending order within subtype. P/NP is whether there is a plasma sequence (yes=1, no=0)/the number of non-plasma sequences;

a Non-plasma analyte available, but mutation found only in plasma;

b 219EQ found in non-plasma analyte and 219E in plasma; 103NS found in non-plasma analyte and 103N in plasma;

c plasma sequence available, but mutation found only in non-plasma analytes (DBS and STP for 219Q; DBS for 219EQ and 221Y);

d plasma sequence not available.

**Figure 2:**
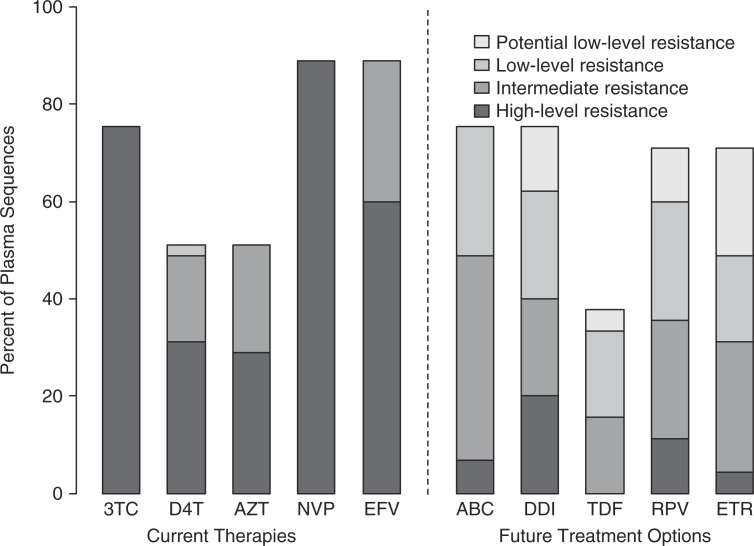
Predicted drug resistance to current ART and future treatment options in treated patients. “Current therapies” to the left of the dashed line, refers to medications taken by participants at the time of the study; “Future therapies” to the right of the dashed line refers to potentially available subsequent second-line RT inhibitor options at the time of the study. Bars show percent of plasma sequences with predicted resistance according to four categories, based on resistance scores available at the Stanford HIV Sequence Database [56]. 3TC, lamivudine; D4T, stavudine; AZT, zidovudine; NVP, nevirapine; EFV, efavirenz; ABC, abacavir; DDI, didanosine; TDF, tenofovir; RPV, rilpivirine; ETR, etravirine.

In plasma sequences, 89% of patients had intermediate-to-high predicted resistance to first-line regimens, 13% for one, 27% for two and 49% for all three drugs; 40/45 (89%) for nevirapine and efavirenz, (34/45) 76% for lamivudine, (22/45) 49% for stavudine, and (23/45) 51% for zidovudine (Figure 2). Sixty percent had intermediate or high-level resistance to subsequent second-line RT inhibitors treatment options, including 11% to all five medications.

Examination of co-occurrence of resistance mutations in this non-B subtype cohort, checked against sequences with at least two resistance mutations in the Stanford Database [56], revealed unique patterns in 11/21 (52%) subtype A sequences, that were not found among 295 subtype A sequences from patients on NRTIs+NNRTI; and 5/21 (24%) that were not found among 10,767 subtype B sequences with the same ART. For subtype D, 4/6 (67%) demonstrated unique patterns compared to 188 subtype D Stanford sequences, one of which (17%) was seen in only one subtype B sequence. The one subtype C sequence had a pattern that was not seen in 1636 subtype C or the subtype B sequences. Additionally, V106M, a subtype-C specific NNRTI mutation [60] was observed in our dataset in one subtype A isolate, observed previously only in one subtype A sequence from South Africa [61].

Combining analytes, 90% had RT-associated mutations, 73% NRTI and 90% NNRTI; 16% had one-class resistance, and 73% dual-class. Five patients did not have evidence for any DR despite treatment failure. This is most likely due to poor medication adherence, though all five did report full adherence.

### Analyte concordance for resistance mutations

A total of 97 mutations (50 NRTI; 47 NNRTI, at 45 positions) appeared in plasma sequences that had non-plasma analyte pairs. Of those, 65/74 (88%) were identified in DBS (39/43, 91% NRTI; 26/31, 84% NNRTI); 17/19 (89%) in DPS (8/8, 100% NRTI; 9/11, 82% NNRTI); and 34/36 (94%) in STP (19/19, 100% NRTI; 15/17, 88% NNRTI). Mutation detection was not significantly different by analyte type.

Additional mutations, not identified in plasma, included three in DBS (2 NRTI; 1 NNRTI); two in DPS (1 NRTI; 1 NNRTI); and six in STP (3 NRTI; 3 NNRTI). Combining non-plasma analytes, of 97 plasma mutations, 84 (87%) were also identified by non-plasma analytes (46/50, 79% NRTI; 38/47, 81% NNRTI); and 10 additional mutations, not identified in plasma, were detected in non-plasma analytes (5 NRTI; 5 NNRTI). Of 23 discordant mutations, 12/13 (92%) in plasma only and 6/10 (60%) in non-plasma analytes only were mixtures (*p*=0.13).

Table 3 provides details on specific mutations discordance among analytes for the seven patients with sequences from four analytes. Of 12 mutations (7 NRTI; 5 NNRTI), 4 (33%) were discordant between plasma and non-plasma analytes, two appearing in DPS only and two in STP only.

**Table 3:** Discordant resistance and non-resistance mutations among patients with sequences from plasma and three non-plasma analytes

			Discordant resistance mutations		Discordant non-resistance mutations^a^				
				
ID^b^	Subtype	Stratum	NRTI (total^c^)	NNRTI (total^c^)	Total^f^	Plasma	DBS	DPS	STP
25	A	Treated	None (0)	None (1)	17	D123N	D123DGNS	D123DN, K154KT, N175DN	D123N, G141EG
4	A	Treated	None (5)	K103S^d^ (3)	16	P140Q		T165R, Q242HQ	
50	DA	Naïve	None (0)	None (0)	23	D121H, K122E, D123S, S162HNR, S I244IV	D121H, K122E, D123S, G141EG, S162R	D121H, K122E, D123S, A158AP, S162HNRS, I244IV	T39IT, P52HP, D110N, D113E, A114S, V118D, L120FL, D121X, K122X, D123X, T131NT, N137IN, S162HN, I244V
51	A	Naïve	D67DN^d^ K219KQ^e^ (2)	E138G^e^ (1)	17	K173AS, R211KR	E169DE, K173AS, R211KR	E169D, K173S, F214FL, L234IL, H235HP	I142N, R143K, M164MV, E169D, F171FL, K173S, I202IM, R211KR, G231D
52	A	Naïve	None (0)	None (0)	15				S68RS, E79DEV, N81KN, R83RS, D86AD
53	A	Naïve	None (0)	None (0)	13				
54	AD	Naïve	None (0)	None (0)	9	R211KR		R211KR	R211KR

Table is sorted by treatment status then by number of total discordant mutations;

a amino acids are shown for each analyte at positions where mutations were found to be discordant in at least one analyte;

b ID<50 match those in Table 2; values>49 were given to naïve patients as to not overlap with Table 2.

c total number of mutations occurring in sequences from all analytes are shown in parenthesis;

d DPS only;

e STP only;

f total number of non-resistance-associated mutations occurring in sequences from all analytes, compared to HIV-1 subtype B. DBS, dried blood spots; DPS, dried plasma spots; STP, ViveST plasma.

### Analyte concordance for whole sequences

In 59 patients with sequences from ≥2 analytes, mean plasma-DBS NA discordance was 1.1% (*n*=33; range 0.4–2.3%; 1.2% for naïves and 1.1% for treated, Table 4). Similar values for plasma-DPS discordance were 1.2% (*n*=15; range 0.3–2.2%; 1.4% naïves and 0.9% treated); 2.0% for plasma-STP discordance (*n*=34; range 0.5–7.1%; 2.2% naïves and 1.2% treated); and 2.3% for plasma-STB discordance (*n*=5; range 0.8–5.3%; 2.3% naïves; no data for treated).

**Table 4:** Nucleic acid discordance between plasma and non-plasma sequence pairs by treatment status and subtype

		DBS	DPS	STP	STB
Total					
	*N*	33	15	34	5
	% Discordance; Range	1.1; 0.4–2.3	1.2; 0.3–2.2	2.0; 0.5–7.1	2.3; 0.8–5.3
Patient stratum					
Treated					
	*N*	17	5	7^a^	0
	% Discordance; Range	1.1; 0.4–2.2	0.9; 0.3–1.3	1.2; 0.7–2.0	–
Naïve					
	*N*	16^b^	10^b^	27	5
	% Discordance; Range	1.2; 0.5–2.3	1.4; 0.4–2.2	2.2; 0.5–7.1	2.3; 0.8–5.3
Treated vs. Naïve	RR (95% CI), *p*	0.9 (0.7, 1.3) *p*=0.70	0.7 (0.5, 1.1) *p*=0.09	0.6 (0.4, 0.8) *p*=0.002	–
Subtype					
A					
	*N*	17	10	18^b^	2
	% Discordance; Range	1.1; 0.4–2.3	1.3; 0.7–2.0	2.0; 0.5–4.7	2.0; 1.4–2.6
D					
	*N*	6	2	5	0
	% Discordance; Range	1.0; 0.4–2.2	1.5; 0.9–2.2	1.0; 0.7–1.6	–
D vs. A	RR (95% CI), *p*	0.9 (0.5, 1.6) *p*=0.64	1.1 (0.8, 1.6) *p*=0.50	0.5 (0.4, 0.7) *p*<0.001	–
Viral load					
Per 1-log unit higher	RR (95% CI) *p*	0.7 (0.6, 0.9) *p*=0.001	0.9 (0.7, 1.4) *p*=0.31	0.9 (0.6, 1.3) *p*=0.59	0.8 (0.5, 1.3) *p*=0.32

Numbers represent mean (range) percent discordance. RR, rate ratios (95% confidence interval) of discordant nucleic acids comparing patient treatment status and subtype by analyte type. “–” means that there were no STB/plasma sequence pairs;

a two missing protease sequences;

b one missing protease sequence. DBS, dried blood spots. DPS, dried plasma spots. STP, ViveST plasma. STB, ViveST blood.

In the seven patients with sequences from four analytes (Table 3), of 110 non-resistance mutations, only one (P140Q) was found in plasma only and 31 in a non-plasma analyte. Of the five discordant plasma-DBS mutations, three were in plasma and two in DBS; of 11 plasma-DPS discordances, two were in plasma and nine in DPS; of 23 plasma-STP discordances, one was in plasma and 22 in STP.

The comparison of plasma-to-non-plasma discordance rates demonstrated that naïve and treated patients had similar rates in DBS, and treated patients had lower rates in DPS (RR=0.7, CI=0.5–1.1, *p*=0.09, Table 4) and STP (RR=0.6, CI 0.4–0.8, *p*=0.002), compared to naïve patients. We found similar discordance rates among subtypes A and D in both DBS and DPS, but subtype D had lower discordance rates in STP compared to subtype A (RR=0.5, CI=0.4–0.7, *p*<0.001). For all analytes, discordance was inversely related to VL (i.e. lower VL had higher discordance), but significantly so only for DBS (RR=0.7 per 1-log higher VL, 95% CI=0.6–0.9, *p*=0.001).

## Discussion

In AMPATH, HIV-1 infected patients demonstrated high subtype diversity, infrequent TDR and high acquired DR with unique mutation patterns upon first-line failure. Virologic failure was identified among ART-experienced patients using WHO guidelines in a setting where VL testing is limited [7]. The enrolment of both ART-naïve and experienced patients and the use of multiple analytes for resistance testing enabled capacity building to conduct resistance studies. In this setting higher VL and a shorter shipment-to-genotyping time were associated with genotyping success.

The HIV-1 subtype distribution at AMPATH in western Kenya is mostly consistent with prior reports from the wider region, where subtypes A, D and C predominate [25, 29, 30, 33, 62]. We identified unique *pol* recombinants in 12%, similar to one Uganda report [63], and higher than prior reports. Identification of inter- and intra-subtype recombination depends on the genomic region and subtyping methods [64–66].

TDR was seen in 1.8% of plasma sequences, a “low” WHO TDR threshold [67]. This is reasonable, given ART introduction to routine clinical care at AMPATH only in 2001 [10]. These low (<5%) TDR estimates contrast with more recent reports of higher-level (6% and 7.4%) TDR from Nairobi, the Kenyan coast and neighbouring Uganda [42, 68]. The TDR increase to 7% combining multiple analytes deserves further attention. Using multiple analytes may be advantageous, and analogous to accumulating sequences over time when estimating resistance, rather than a cross sectional assessment [69]. Whether non-plasma analytes are more sensitive in detecting TDR deserves further study. The WHO SDRM list was designed specifically to account for subtype diversity [57] and the high (14%) TDR based on the IAS–USA mutation list emphasizes the importance of using a reference adapted to global subtypes. Despite recent reports [70], we found no difference in TDR between higher and lower CD4 groups or between subtypes, although these conclusions are limited due to small sample-sizes.

Resistance mutations were found in 91% of patients failing a first-line regimen, at the high end of reports from other regional settings (60–80%) [4, 46, 71]. Similar high resistance rates were reported in Burnt-Forest, a rural AMPATH clinic [34]. These high rates may be related to adherence, lack of virologic monitoring and treatment failure misclassification by immunologic criteria, each of which contributes to resistance accumulation [7, 72].

Our results are relevant even with samples collected in 2006–2007 and WHO guidelines to phase out stavudine and phase in tenofovir [37]. Implementation of such guidelines is slow and resistance can be transmitted and relevant for tenofovir-based regimens. Similarly, there are significant implications of high resistance levels; 76% 2-class resistance, 47% ≥5 mutations and 60% intermediate–high predicted resistance to future ART. These findings provide a strong rationale for the use of a boosted PI in second-line ART with an active NRTI to limit transmission and continued accumulation of resistance. Continued monitoring of resistance as well as routine VL testing are important, to enable early failure identification and limit resistance accumulation [73]. No differences in resistance development were observed among subtypes, although larger studies suggest such differences [71, 74, 75], justifying further research. Unique resistance patterns in diverse subtypes identified here may support subtype-specific and host-specific resistance pathways, though other viral, host and environmental factors should be considered.

Non-plasma analytes have been explored for resistance testing in RLS, mostly for their cost and simplicity [14, 17, 76]. This is the first report of comparison of four different non-plasma analytes to plasma. Though these pilot results demonstrated lower genotyping yield in non-plasma analytes, we demonstrate their feasibility in these “real life” laboratory settings. The relatively low levels of successful genotyping may be ascribed to potential sample mishandling, freeze thawing, prolonged exposure to higher temperature and duration between collection and assay. Reduced rates of reverse transcription and amplification of HIV RNA were also documented in a study from Asian and African sites which compared storage temperatures and duration [77]. Lower-yield results with field-plasma on ST resemble a recent report [54], however the higher success rates of genotyping using frozen-plasma (highest among non-plasma analytes), under controlled drying conditions, more closely resemble previously published success rates for VL from ST [16]. These findings highlight the importance of complete sample drying with any ST devices. Furthermore, the ST colour indicator provides an additional cost-benefit in preventing unnecessary reagent loss. With higher ART and VL monitoring access and use of non-plasma analytes [78, 79], the need to increase sensitivity, yield and local laboratory capacity to use such analytes will rise [80].

High (93–99%) full sequence concordance was demonstrated between plasma and non-plasma analytes, confirming prior results with DBS [17, 76] and one study with ST [54], and providing support for non-plasma analytes for resistance testing. Our results confirm recent findings of an inverse relationship between VL and sequence discordance [81]. Most of the resistance mutations that were not identified in plasma were seen in DPS and STP rather than DBS. Such findings, as well as resistance mutations observed in plasma but not non-plasma analytes in seven patients, are relevant to global DBS resistance surveillance recommended by WHO, and additional research is needed to better understand this phenomenon and its implications. Resistance mutation concordance between plasma and non-plasma analytes was slightly lower (88–94%), with most discordances being mixtures. Unique data from seven patients with sequences from four analytes even demonstrated 33% discordant resistance mutations. Whether these findings are related to different sensitivities of filter analytes to hold stable DNA and/or RNA, the different sequencing methodology used for ST, or variables such as sampling, extraction or amplification methods or VL, remains to be determined.

Our findings from AMPATH in western Kenya are relevant to HIV care and resistance monitoring in other RLS, where the rising treatment roll-out can lead to increasing selection and transmission of resistance. The provision of prevention, treatment and clinical care may benefit from on-going examination of resistance and sequence diversity, to avoid evolution of extensive resistance, particularly in settings with limited ART regimens. As treatment programmes expand to meet WHO guidelines for increased VL monitoring [82], the need for resistance testing will increase as well, and better low-cost methods are needed. Challenges to the currently recommended plasma genotyping include cost and complexity of on-site phlebotomy, centrifugation and separation, and maintenance of a transport cold-chain. These challenges may be overcome by the use of analytes such as DBS, and ST. This evaluation of multiple analytes for genotyping in a RLS with subtype diversity and with barriers like minimal infrastructure, demanding transportation requirements, and high temperatures and humidity, demonstrates feasibility and further optimization needs. This study also underscores the potential advantage of using multiple analytes in resistance determination. Although the demonstrated sequence concordance among analytes is encouraging, their utility in research and clinical care will require larger scale evaluation of feasibility and effectiveness.

It is important to recognize that this was a pilot study, reflecting on its small sample size, limiting our ability to robustly examine resistance, subtype effects and plasma-non-plasma analyte concordance, or draw inference to other settings. Yet it is the first examination of HIV resistance and diversity in a large clinic in western Kenya. Other limitations include use of early DBS and ST versions, reducing genotyping yield and usage of different genotyping methods for ST versus other analytes.

## Conclusions

High levels of HIV diversity and resistance in multiple subtypes were observed in western Kenya. Although new antiretroviral agents and classes are in development, resistance remains a major challenge to treatment, particularly in RLS with a high burden of infection, diverse HIV variants, increasing treatment access, low treatment monitoring capacity and limited medications. Lower cost, robust analytes and assays to monitor resistance are important and useful in maintaining the benefit of ART.
